# Fine-mapping of candidate region in Amish and Ashkenazi families confirms linkage of refractive error to a QTL on 1p34-p36

**Published:** 2009-07-17

**Authors:** Robert Wojciechowski, Joan E. Bailey-Wilson, Dwight Stambolian

**Affiliations:** 1 Statistical Genetics Section, Inherited Disease Research Branch, National Human Genome Research Institute (NIH), Baltimore, MD; 2 Departments of Ophthalmology and Genetics, University of Pennsylvania, Philadelphia, PA

## Abstract

**Purpose:**

A previous genome-wide study in Orthodox Ashkenazi Jewish pedigrees showed significant linkage of ocular refraction to a Quantitative Trait Locus (QTL) on 1p34-36.1. We carried out a fine-mapping study of this region in Orthodox Ashkenazi Jewish (ASHK) and Old Order Amish (OOA) families to confirm linkage and narrow the candidate region.

**Methods:**

Families were recruited from ASHK and OOA American communities. The samples included: 402 individuals in 53 OOA families; and 596 members in 68 ASHK families. Families were ascertained to contain multiple myopic individuals. Genotyping of 1,367 SNPs was carried out within a 35cM (~23.9 Mb) candidate QTL region on 1p34-36. Multipoint variance components (VC) and regression-based (REG) linkage analyses were carried out separately in OOA and ASHK groups, and in a combined analysis that included all families.

**Results:**

Evidence of linkage of refractive error was found in both OOA (VC LOD=3.45, REG LOD=3.38 at ~59 cM) and ASHK families (VC LOD=3.12, REG LOD=4.263 at ~66 cM). Combined analyses showed three highly significant linkage peaks, separated by ~11cM (or 10 Mb), within the candidate region.

**Conclusion:**

In a fine-mapping linkage study of OOA and ASHK families, we have confirmed linkage of refractive error to a QTL on 1p. The area of linkage has been narrowed down to a gene-rich region at 1p34.2-35.1 containing ~124 genes.

## Introduction

Ocular refraction is a complex phenotype that is affected by a host of environmental and biological influences. A number of studies in a variety of populations have shown refractive error to be highly heritable [1-5]. Almost twenty genetic loci for myopia or refractive error have been identified in linkage studies, but few have been reliably reproduced in independent populations (see e.g., the Online Mendelian Inheritance in Man [OMIM] database for a complete list, and Tang et al. [6] for a good review). One reason for this difficulty in confirming loci for refraction is the inherently complex nature of refractive error, wherein multiple interacting genes and environmental factors likely contribute to differential eye growth and refractive regulation. Moreover, many myopia loci were mapped in highly-selected families that aggregated severe forms of myopia, which may have different genetic etiologies than more common types of refractive errors [7-9]. Finally, genetic linkage studies generally lack the statistical power to detect loci of small effect, and coding of refraction phenotypes may be inconsistent across studies, making comparisons and generalizations troublesome. In an attempt to address some of these issues, the Myopia Family Study (MFS) was developed to systematically search for the genetic causes of refractive errors in extended families from distinct ethnic groups.

The first genetic linkage studies of refractive phenotypes involved an X-linked syndromic form of myopia termed Bornholm disease [10]. Later, Young et al. successfully mapped severe myopia loci in a small number of extended families to 18p11.31 [8] and 12q21-23 [7] using parametric linkage methods. Parametric statistics were well-suited to detect linkage in these highly-selected families in which an underlying genetic model–in this case autosomal-dominant–could be reasonably assumed. More recently, linkage studies have also focused on more prevalent refractive errors, such as low-to-moderate myopia [11-13] and ocular refraction, as a quantitative phenotype [14-17]. The quantitative trait locus (QTL) linkage analyses of refractive error were conducted in both population-based cohorts [15,16] and selected samples of families [14,17,18]. These various sampling and analytical strategies have yielded numerous loci linked to refraction traits. Nevertheless, although some of these mapped linkage regions were successfully reproduced in independent samples [12,16,18-21], many more loci have not been replicated [22].

The Myopia Family Study is comprised of families from four American ethnic groups: Orthodox Ashkenazi Jewish (ASHK); Old Order Amish (OOA); African American (AA); and Caucasian (CAU). Thus far, analyses of the AA families have shown genome-wide significant linkage of refraction to a QTL on chromosome 7p15 [14]. This region has also been seen in French families with high myopia [23] and suggested independently in a population-based cohort of sibships from the Beaver Dam Eye Study [16]. Analyses of ASHK families have identified a locus for myopia on chromosome 22q12 [20]. This finding has since been confirmed by investigators from the Beaver Dam study [16], and replicated in an independent cohort of ASHK families from the MFS [20]. These results suggest that, while refractive errors undoubtedly have multifactorial genetic and environmental etiologies, at least some genetic polymorphisms involved in refractive variation may be shared across populations.

In an analysis of the Ashkenazi Jewish sample of the MFS, we previously reported genome-wide significant linkage of ocular refraction to a novel QTL at 1p34-36 [17]. A subsequent meta-analysis of all MFS families showed no evidence of linkage to this region in OOA, AA, or CAU participants [18]. This lack of corroboration could be due to several possibilities: allele frequency differences between ethnic groups; locus heterogeneity; false positive results in the original analysis; and/or due to a lack of statistical power for replication. We subsequently genotyped a dense map of SNP markers within the area of linkage in families from the ASHK and OOA cohorts (see methods) in an attempt to replicate the findings in a fine-mapping linkage study in OOA families, and narrow down the region of interest in both the OOA and ASHK for subsequent positional cloning experiments. This manuscript presents results from fine-mapping linkage analyses designed to address these aims. We report replication of chromosome 1 linkage results. In addition, using a 1 LOD cutoff as boundaries for the area of linkage in both ASHK and OOA families, we narrow down the location of the QTL to a 10 Mb area at 1p34.2 to 1p35.2.

## Methods

Family recruitment and selection criteria have been reported elsewhere and are summarized here [12,13]. Briefly, ASHK and OOA participants were recruited into the Myopia Family Study primarily from the Lakewood, NJ (ASHK) and Lancaster County, PA (OOA) areas. All participating individuals were of either Old Order Amish or Orthodox Ashkenazi Jewish cultural/religious heritage (individuals of Sephardic Jewish origin and their offspring were not included in the study). For the OOA, myopic individuals were identified through community liaisons and their families were invited to participate in the study; ASHK participants were asked to participate through mass mailings sent to all known Orthodox Jewish families residing in Lakewood township, NJ. In order to be eligible for inclusion in the study, a nuclear family had to contain only one myopic parent and at least one myopic offspring. These criteria were established to enhance selection of autosomal-dominantly transmitted myopia within families. Larger pedigrees were then formed by extending nuclear families through first- and second-degree relatives. Extended families were then selected for the linkage study if 1) there was at least one affected pair of relatives besides a single parent-offspring pair and 2) biological specimens were available for at least these affected individuals. The study protocol adhered to the tenets of the Declaration of Helsinki and was approved by the University of Pennsylvania and the National Human Genome Research Institute, National Institutes of Health institutional review boards.

All participants underwent a comprehensive eye examination including: medical and ophthalmic histories; visual acuities; slit lamp examination; Goldmann applanation tonometry; fundoscopy; and objective and manifest refraction. Individuals under 41 years of age also received cycloplegic refraction using 0.5% cyclopentolate or 1% tropicamide. When participants could not be examined at our study clinics, we attempted to obtain their most recent ocular examination records from their eye care providers. The quantitative phenotype, ocular refraction, was defined as the manifest spherical equivalent refractive error, averaged between the eyes. Cycloplegic refractive error was used to define ocular refraction for participants under age 41, or when available from ocular examination records. Because the initial recruitment strategy focused on myopia as a binary trait, individuals under age 21 with a refractive error between 0 and -1 D in any principal meridian were classified as "unknown" and were excluded from the study. Also ineligible were individuals who had ocular or systemic conditions that could affect ocular refraction or significantly compromise the accuracy of refractive measurements. These conditions included: a history of prematurity; connective tissue disorders; poorly controlled diabetes mellitus; keratoconus; cataract; corneal opacities; and ocular syndromes in which myopia is a defining or common feature.

Individuals selected for fine-map SNP genotyping were drawn from participating ASHK and OOA families in the MFS. Families were selected based on informativeness for linkage, DNA quality and availability, and sample size constraints. Most of these families were also included in previously-reported genome-wide linkage studies [12,13,17,18,20]: 44 ASHK (out of 68) and 51 (out of 53) OOA families in the current analysis overlapped with pedigrees from previous genome-wide linkage scans of ocular refraction [17,18]. Four ASHK and 10 OOA families from the original genome-wide linkages were not included in the fine-mapping for the reasons given above, generally because these families could contribute virtually no information about linkage.

### Marker selection

All SNPs were chosen a priori by the authors to provide sufficient coverage of a linkage area identified in a previous quantitative-trait analysis of a larger ASHK sample [17]. The linkage peak was located at ~49 cM on chromosome 1p36 between microsatellite markers *D1S552* and *D1S1612*, and the broadly-defined linkage area spanned roughly 35 cM or 23.9 Mb (from 17.5 to 41.4 Mb) at 1p36.13-1p34. Our final candidate region for fine-mapping extended from 18 Mb to 44.2 Mb.

Coverage within the candidate linkage area was obtained by identifying haplotype tagging SNPs from the European population (CEU) in the Human International HapMap consortium [24,25]. Tagging SNPs were picked using the two-SNP aggressive tagging algorithm of the online Tagger software server [26] so as to cover common SNPs (MAF >=0.25) at a minimum r^2^ of 0.7. In addition, coverage gaps larger than 200 Kb were filled-in with non-tagging polymorphic SNPs. We also included for genotyping all known functional SNPs (i.e., missense and nonsense mutations) with MAF of at least 0.1. In total, 46 unique functional SNPs, with average heterozygosity=0.43, were genotyped.

### DNA Extraction and Genotyping

Venipuncture was used to collect peripheral blood from participating family members. High molecular weight genomic DNA was extracted from the blood samples with a Puregene DNA purifying kit (Gentra Systems, Inc; Minneapolis, MN). The purified DNA was then stored in a refrigerated DNA repository under a unique sample code. Custom SNP genotyping was carried out at the Center for Inherited Disease Research (Johns Hopkins Medical Institutions, Baltimore, MD) on an Illumina BeadLab system (Illumina, Inc., San Diego. CA) using GoldenGate chemistry.

### Quality control and data cleaning

A total of 1,367 SNPs were genotyped in the chromosome 1 candidate region. Of these, 127 (9.2%) were excluded from analysis because of potential genotyping errors: 45 because of unreliable raw intensity scores or call rates below 95%; 61 due to atypical intensity clustering patterns; and 21 were found to be monomorphic. Also removed from analysis were SNPs that departed significantly from expected Hardy-Weinberg proportions (p<0.01) in either ASHK (13 SNPs) or OOA (4 SNPs) founders. These quality control measures resulted in 1,227 and 1,236 high quality SNPs being available for analysis in ASHK and OOA families, respectively.

The programs PEDCHECK [27], PEDSTATS [28], and RELCHECK [29,30] were used to check for Mendelian transmission inconsistencies and confirm putative pedigree relationships. All Mendelian and relationship errors were corrected prior to analysis. When multiple Mendelian transmission errors could not be reconciled with pedigree structures, incompatible individuals were removed from the dataset. Large pedigrees were split to accommodate the default memory limits of multipoint analysis in the program MERLIN [31] (i.e., 24 bits for Lander-Green algorithm), making sure that no individuals were duplicated across pedigrees.

### Statistical analysis

The statistical package MERLIN [31] (version 1.1.2) and its subroutine MERLIN-REGRESS [32] were used to perform multipoint quantitative trait locus (QTL) linkage analyses. Linkage statistics were estimated using all SNPs that passed our quality-control filtering. Because these tagging SNPs were chosen to minimize between-marker linkage disequilibrium, an increase in type-1 error rates was deemed unlikely. However, to guard against this possibility we repeated our analyses using a randomly-chosen subset of 198 SNPs spaced at least 100 Kb apart (average spacing 133 Kb).

Both variance components (VC) and regression-based (REG) QTL linkage analyses were performed on logarithmic transformations of the spherical equivalent refractive error. Allele frequencies were estimated using the maximum-likelihood method in MERLIN. All calculations were performed separately for ASHK and OOA families, and after combining families from both populations. The modified Haseman-Elston approach implemented in MERLIN-REGRESS [32] requires that the trait mean, variance, and heritability of the underlying populations be pre-specified. Although these parameters are unknown in Orthodox Ashkenazi Jews and the Old Order Amish, we estimated them using previous epidemiological studies in related populations [4,33]. We have also shown, in previous reports, that estimates of linkage statistics in MERLIN-REGRESS are stable under reasonable population parameter specifications [17]. However, because population means and variances of refractive error are thought to be significantly different between Orthodox Ashkenazi Jews and the Old Order Amish, combined REG and VC analyses were performed after standardizing refraction errors to the respective empirical means and variances of both groups. Hence, values for mean and variance parameters were set to 0 and 1, respectively, for combined REG linkage analyses, while the heritability for the combined groups was set to 0.60.

## Results

### Population characteristics

Sample characteristics of OOA and ASHK families are presented in Table 1. There were 402 individuals in 53 OOA pedigrees including: 182 male; 220 female; 127 founders; and 278 non-founders. A total of 328 (81.6%) OOA participants were genotyped. OOA families averaged 7.6 persons per family and 2.1 generations. The mean spherical equivalent refractive error among all OOA participants was -1.61D (sd=2.72) and 184 (54.2%) of 332 individuals with known phenotypes had myopia of at least 1D in both eyes.

**Table 1:** Sample characteristics.

**Population**	**Families**	**n**	**Family size (range)**	**Generations (range)**	**Genotyped (%)**	**Male (%)**	**Female (%)**	**Founders (%)**	**Mean age (range)**	**Number myopic (%)**	**MSE (SD)**
Old Order Amish (OOA)	53	402	7.58 (3-25)	2.09 (2-4)	328 (82)	182 (45)	220 (55)	124 (31)	36.7 (9-85)	184 (54.2)	-1.61 (2.72)
Ashkenazi Jewish (ASHK)	68	596	8.76 (4-17)	2.46 (2-4)	527 (88)	308 (52)	288 (48)	181 (30)	40.3 (9-94)	414 (76.3)	-3.56 (3.31)
Combined:	121	998	8.25 (3-25)	2.30 (2-4)	855 (86)	490 (49)	508 (51)	305 (31)	38.8 (9-94)	598 (68.4)	-2.75 (3.23)

Myopia is defined as a manifest (or cycloplegic) refraction ≤-1 D in all four principal meridians. The proportion of myopic individuals (%) estimate excluded unknown phenotypes from the denominator. MSE=Mean Spherical Equivalent refractive error.

The ASHK sample contained 68 families and 596 individuals (308 males and 288 females). The average pedigree for the ASHK sample was comprised of 8.8 individuals in 2.5 generations. Genotypes were obtained for 527 (88.4%) ASHK participants. The mean refractive error among all ASHK was -3.56D (sd=3.31) and 414 (76.4%) of 542 subjects with known phenotypes were categorized as myopic (≤-1D in both eyes).

OOA and ASHK participants showed significant differences in both the mean refractive error and the proportion of myopic participants. These disparities are likely due to differences in the distribution of refractive error in the underlying populations: whereas the Old Order Amish are thought to be at low risk for myopia [4], the prevalence of myopia among Orthodox Jewish communities is considerably higher [33,34]. However, it is important to note that these families were selected for linkage studies of myopia and thus are not representative of refractive error distributions in their populations.

### Marker statistics

The average call rate of all SNPs was 0.96. After excluding SNPs with a call rate of 0, the call rate was 99.9%. The mean minor allele frequency of non-monomorphic SNPs was 0.369 (sd=0.089), the average heterozygosity was 43.9% (sd=7.06) and the mean between-SNP spacing was 21 Kb (range 0.1-31.7).

### Linkage analysis

**Figure 1:**
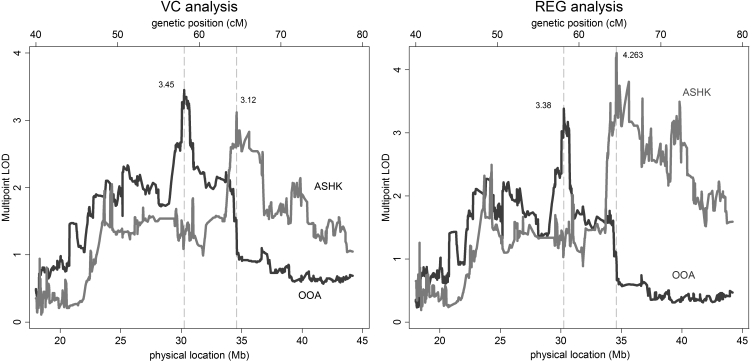
Multipoint variance-components (left) and MERLIN-REGRESS (right) LOD scores for Old Order Amish (OOA) and Ashkenazi Jewish (ASHK) families. Locations of linkage peaks are indicated by vertical lines. Physical locations of SNP markers (bottom axis, in Mb) were determined using the NCBI dbSNP reference map, build 123. Genetic map positions (top axis, in cM) were obtained through the Rutgers Combined Linkage-Physical Map of the Human Genome. Because the relationship between physical position and genetic maps are non-linear, genetic positions in the figure are approximate.

**Figure 2:**
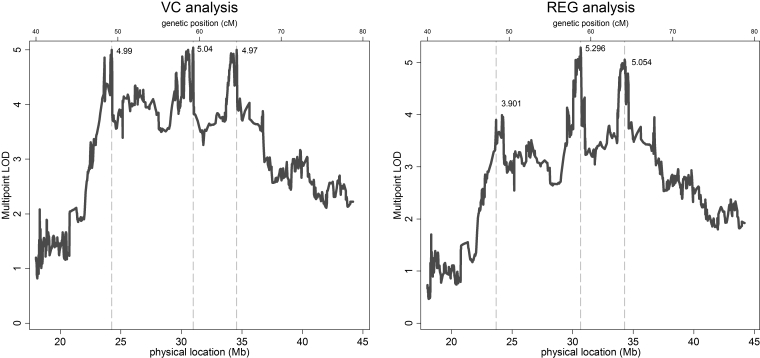
Multipoint variance-components (left) and MERLIN-REGRESS (right) LOD scores for a combined analysis of OOA and ASHK families. Locations of local linkage peaks are indicated by vertical lines. Physical locations of SNP markers (bottom axis, in Mb) were determined using the NCBI dbSNP reference map, build 123. Genetic map positions (top axis, in cM) were obtained through the Rutgers Combined Linkage-Physical Map of the Human Genome. Because the relationship between physical position and genetic maps are non-linear, genetic positions in the figure are approximate.

Results from multipoint VC and REG linkage analyses for ASHK and OOA families are presented in Figure 1. The maximum multipoint VC LOD scores for mean spherical equivalent refractive error were: 3.45 (p=0.00003) at ~59 cM (or 30.2 Mb) for OOA; and 3.12 (p=0.00007) at ~66.4 cM (or 34.6 Mb) for ASHK. For REG analyses, the maximum LOD scores were: 3.38 (p=0.00004) at ~59.7 cM (or 30.25 Mb) for OOA; and 4.263 (p<10^-5^) at ~66.4 cM (or 34.59 Mb) for ASHK families. The 1-LOD support intervals for VC and REG analyses of the OOA sample extend from rs212306 and rs9426315 at 1p35.3 (~59 cM), to rs471202 and rs6687223 at 1p35.2 (~60 cM); and the 1-LOD support regions in ASHK spanned from rs524787-rs549048 (1p35.1, ~65 cM) to rs704784 (1p34.2, 73 cM). The 2-LOD support intervals for VC analyses extended from 51.5 to 66.5 cM in the OOA, and between 53.9 and 62.7 cM for ASHK. Results from QTL multipoint linkage analyses after combining families from both populations are shown in Figure 2. Three linkage peaks were identified in the VC analysis: LOD= 4.99 at ~55 cM (24.3 Mb); LOD=5.04 at ~62 cM (31 Mb); and LOD=4.97 at ~66.5 cM (34.6 Mb). Three local linkage peaks were also found in the REG analyses: LOD=3.901 at ~54 cM (23.7 Mb); LOD=5.296 at ~60.6 cM (30.7 Mb); and LOD=5.054 at ~66 cM (34.3 Mb). All local linkage signals in the combined analysis were highly statistically significant (all p<10^-5^).

**Figure 3:**
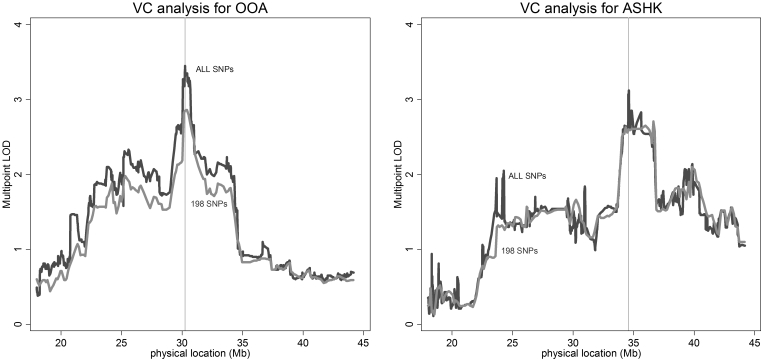
Multipoint variance-components linkage LOD scores for OOA (left) and ASHK (right) for two different SNP densities. ALL SNPs analyses used all markers that passed quality control measures (1,236 for OOA and 1,227 for ASHK); 198 SNPs analyses used a subset of 198 markers spaced at least 100 Kb apart (mean inter-marker distance = 133 Kb). Locations of linkage peaks using ALL SNPs are indicated by vertical lines.

Analyses using a subset of 198 SNP markers yielded similar linkage profiles, although the maximum LOD scores were attenuated relative to analyses with the full marker set: the maximum VC LOD score was 2.86 (p=0.00014) for OOA; and 2.71 (p=0.0002) for ASHK (see Figure 3 for VC results). The maximum REG LOD score using the restricted marker set was 2.55 (p=0.0003) for OOA and 3.98 (p=0.00001) for ASHK. The following discussion will mainly focus on linkage results using all markers.

## Discussion

Using an independent sample of Old Order Amish families, we have confirmed linkage of a candidate region for ocular refraction, previously mapped to a locus on 1p34-p36 in Ashkenazi Jewish families. In the current study, the maximum multipoint LOD scores of 3.45 (p=0.00003) for VC, and 3.382 (p=0.00004) for REG are highly significant in the OOA. Even when using the most conservative test, namely the analyses of OOA using only the 198 SNP markers with no intermarker LD, the p-values were 0.00014 for VC and 0.0003 for REG which satisfy the well-known criteria for linkage replication (p ≤0.01) proposed by Lander and Kruglyak [35].

In a genomewide linkage study of ASHK Jewish families, Wojciechowski et al. [17] first reported genome-wide significant linkage of ocular refraction to a broad region spanning from 1p34 to 1p36; and their peak multipoint linkage signal was seen between microsatellite markers *D1S552* and *D1S1622* at 1p36.13-35.3. In the present study, the area of maximum evidence for linkage in OOA families was centered at markers rs1095026-rs10915029 on 1p35.2 (in both VC and REG analyses). This signal coincides with results from the initial genomewide screen in ASHK. Not surprisingly, the present study also shows evidence of linkage of refractive error to this region in ASHK families. This result was expected since most of the same families from the initial genomewide study [17] were also used in the current analysis. Nevertheless, our fine-mapping analyses (with higher information content in the region and additional families) provide further evidence that the strong linkage signal identified using microsatellite markers was not simply due to chance allele sharing in a region with low linkage information. In the current analysis of ASHK families, the maximum LOD scores were found at rs1016091 (1p34.3) for both VC (LOD=3.12 p=0.00007) and REG (LOD=4.263, p=0.0000049) analyses, with the 1-LOD confidence regions spanning 1p35.1 to 1p34.2.

Even though most of the ASHK participants from the original genomewide scan were used in the current study, fine-mapping linkage signals of ASHK families were further from the original ASHK linkage peak than results from the OOA sample. This is not surprising since localization of linkage peaks can be affected by a variety of factors including statistical variation, and a number of sample-specific factors [36-38] (i.e., new families with a different mixture of linked and unlinked families, differing penetrance and heritability due to allelic heterogeneity at the same locus, meiotic recombination events, map precision, marker informativeness, etc.) . Moreover, a previous genomewide scan of OOA families showed little evidence of linkage of ocular refraction to chromosome 1 (REG LOD=0.996 at 86 cM). This discrepancy can be attributed to the sparseness of microsatellite markers used in the whole-genome study, and the resulting comparatively low marker information content. The low information content in the initial study was one of the motivating factors for the fine mapping performed in the OOA here. In the present analysis, linkage peaks in the OOA and ASHK families were separated by approximately 7 cM. Although the 1 LOD support intervals are separated by 3-5 cM, the 2 LOD support intervals overlap considerably (Figure 1). Given the inherent imprecision in localizing genetic loci for complex traits in genetic linkage studies [37,38], this result is consistent with the presence of a single QTL for ocular refraction in this region. Nonetheless, the possibility of two distinct QTLs contributing to refraction variation among OOA and ASHK families cannot be ruled out.

Compared to single population analyses, our combined analyses of OOA and ASHK families yielded greater evidence for linkage across the entire candidate region (maximum LOD=5.04 for VC; 5.296 for REG). However, in the combined analysis, three distinct linkage peaks can be seen from ~50 to ~65 cM (Figure 2). This may be simply a statistical artifact that resulted from the merging of genetically heterogeneous subgroups. It is also possible that the three peaks are due to locus heterogeneity, wherein more than one locus in the region is linked to the phenotype. However, statistical artifact is more likely than the presence of three refractive error genes in this region. Absent a causative gene(s) or polymorphism(s) for variations in refractive error, it may not be possible to distinguish between these possibilities. Further study would be required to make that determination.

Our results suggest that a single locus may account for variations of refractive error in both OOA and ASHK families. Given the ubiquity of refractive errors in human populations, a genetic locus that is involved in variation of refractive error in different ethnic subgroups can be expected, especially since linkage analysis can detect such a locus even if different ancestral alleles are involved in the different populations or if the same variant alleles are present but at differing frequencies in the different groups. Moreover, both OOA and ASHK American populations are descendents of European migrants to the North American continent within the last few centuries [39,40], suggesting a (relatively) recent phylogenetic separation between the groups. Nevertheless, the OOA and Orthodox ASHK are somewhat culturally isolated, largely endogamous, societies; and significant demographic and socio-cultural differences exist between these groups. In fact, genetic clustering analyses of our study participants showed that individuals could be classified into OOA or AMISH groups with high confidence using a limited number of markers (data not shown).

The distribution of refractive errors differs significantly between OOA and Orthodox ASHK ethnic groups. While Orthodox Jewish groups suffer from high rates of myopia (especially among men) [33,34], the prevalence of myopia is thought to be low among the OOA [4]. This is likely due to widely different exposures to environmental and behavioral risk factors for myopia between these groups. Jewish Orthodoxy emphasizes an intense education in religion and ethics from an early age and, for men, religious scholarship is required throughout life. The OOA, on the other hand, live agrarian lifestyles, eschew technology and oppose any forms of higher education. Because of these disparities in environmental exposures (in addition to inherent population genetic differences), we took care to account for these differences in the design, analysis and interpretation of this linkage study. First, all initial analyses were conducted separately for the OOA and ASHK, limiting the likelihood of bias in our results. Second, phenotypes in combined analyses were normalized within distinct subgroups a priori. Otherwise, the distributional and independence assumptions on which linkage statistics are based could have been violated. Even after such normalization, however, departures from Hardy-Weinberg proportions and imprecise allele frequency estimates could invalidate allele sharing calculations required in estimating LOD scores in combined analyses. Finally, rather than using a binary trait, such as myopia, in our analyses, we analyzed refractive error as a continuous phenotype. In addition to some statistical advantages inherent in quantitative trait linkage statistics, they may be more appropriate for between-group comparisons when distributional differences exist between groups. For instance, quantitative linkage methods assess variations within populations whereas arbitrary thresholding of refractive error to define myopia may not apply equally to OOA and ASHK individuals. Hence, our results suggest that a quantitative trait locus at 1p34.2-35.3 may account for variations in refractive errors in both the OOA and AMISH despite widely divergent underlying trait distributions. This can occur if both genes and environment contribute separately to refractive error regulation and/or if the frequency of alleles differs between the two populations at this quantitative trait locus. In the former model, environmental and behavioral risk factors would cause a shift in the overall distribution of refractive errors towards myopia, while various alleles at a gene (or genes) would account for variations of refractions within this range. This hypothesis is supported by evidence from a number of heritability studies, which consistently show high heritability estimates across ethnic groups with varying prevalences of myopia [1,3-5,41] (though Mendelian forms of myopia or hyperopia may be less subject to environmental influence).

Using a 1-LOD drop as a cutoff, the area of linkage in the present study spans from rs212306 (the telomeric boundary in OOA) to rs704784 (the centromeric boundary in ASHK). To our knowledge, no other studies have reported linkage of either myopia or refractive error to this region. The linkage region in the current analysis spans approximately 10 Mb and contains 124 human genes in the NCBI RNA reference sequence collection (RefSeq). To date, 5 of these genes (fatty acid binding protein 3 [*FABP3*], gap junction protein, alpha 4 [*GJA4*], gap junction protein, alpha 3 [*GJA3*], glutamate receptor, ionotropic, kainate 3 [*GRIK3*], and solute carrier family 31 (copper transporters), member 1 [*SLC3A1*]) have been reported in human association studies [42], three of which have shown positive associations to disease phenotypes. However, no genes in the candidate region have, as of yet, been reported as being associated with refraction or other ocular traits in humans. Two mouse orthologs of human genes within the linkage area (collagen, type VIII, alpha 2; and neurochondrin) have been shown to influence ocular phenotypes in experimental studies in mouse [43] –though neither of these seem to be particularly strong candidates for refractive error control.

In order to follow-up our findings and identify causal polymorphisms for refractive error variation, we are conducting family-based association analyses of SNP markers. These analyses will help identify either causal polymorphisms or markers strongly correlated with causal genetic variations (i.e., via linkage disequilibrium). In the future, association analyses of additional SNP and copy-number polymorphisms in the region may help to identify the DNA variants responsible for this linkage signal.

### Summary

In a fine mapping study in Orthodox Ashkenazi and Old Order Amish families, we have confirmed linkage of refractive error to a quantitative trait locus on 1p and have narrowed the region of interested to a ~10 Mb area spanning 1p34.2-35.1. Given that linkage was found in two independent, culturally and genetically isolated, groups, it is probable that the underlying genetic cause is a single genetic locus with variant alleles of large effect that can be detected in both these ethnic groups. It is reasonable to expect that such a locus would also have variant alleles that influence refractive error in other European-derived populations.
